# Skin microbiome alterations in upper extremity secondary lymphedema

**DOI:** 10.1371/journal.pone.0283609

**Published:** 2023-05-17

**Authors:** Adana-Christine Campbell, Teng Fei, Jung Eun Baik, Hyeung Ju Park, Jinyeon Shin, Kevin Kuonqui, Stav Brown, Ananta Sarker, Raghu P. Kataru, Babak J. Mehrara

**Affiliations:** 1 Division of Plastic and Reconstructive Surgery, Memorial Sloan Kettering Cancer Center, Department of Surgery, New York, NY, United States of America; 2 Department of Epidemiology and Biostatistics, Memorial Sloan Kettering Cancer Center, New York, NY, United States of America; Universidad San Francisco de Quito, ECUADOR

## Abstract

Lymphedema is a chronic condition that commonly occur from lymphatic injury following surgical resection of solid malignancies. While many studies have centered on the molecular and immune pathways that perpetuate lymphatic dysfunction, the role of the skin microbiome in lymphedema development remains unclear. In this study, skin swabs collected from normal and lymphedema forearms of 30 patients with unilateral upper extremity lymphedema were analyzed by 16S ribosomal RNA sequencing. Statistical models for microbiome data were utilized to correlate clinical variables with microbial profiles. Overall, 872 bacterial taxa were identified. There were no significant differences in microbial alpha diversity of the colonizing bacteria between normal and lymphedema skin samples (p = 0.25). Notably, for patients without a history of infection, a one-fold change in relative limb volume was significantly associated with a 0.58-unit increase in Bray-Curtis microbial distance between paired limbs (95%CI = 0.11,1.05, p = 0.02). Additionally, several genera, including *Propionibacterium* and *Streptococcus*, demonstrated high variability between paired samples. In summary, we demonstrate high compositional heterogeneity in the skin microbiome in upper extremity secondary lymphedema, supporting future studies into the role of host-microbe interactions on lymphedema pathophysiology.

## 1. Introduction

Secondary lymphedema (LE) is a chronic condition of the lymphatic system that is characterized by fibroadipose tissue deposition, chronic inflammation, and, in some cases, recurrent infections [1, 2]. In fact, nearly 40% of patients with LE develop recurrent cellulitis and lymphangitis requiring antibiotic treatment and hospitalization [3, 4]. In some cases, LE-related infections can be severe, resulting in sepsis and even death [5]. For example,92% of the 165,055 LE-related hospital admissions in the US between 2012–2017 were for treatment of cellulitis and had an associated inpatient mortality of 0.03% [6].

Patients with secondary LE have impaired immune responses to bacterial and viral antigens, making recurrent infections more likely; however, the mechanisms that underlie this increased risk remain largely unknown [7, 8]. In the past ten years our lab and others have shown that chronic T-helper 2 (TH2) immune responses are an important pathological response in LE and these responses are known to cause barrier disruption in other chronic inflammatory skin disorders [9, 10]. Impaired barrier function may thus provide a port of entry for bacteria since the skin is an important defense against infections [11]. In addition, the accumulation of protein-rich fluid in LE provides an optimal environment for bacterial colonization [12].

Alterations in the skin microbiome are associated with cutaneous skin disorders, such as atopic dermatitis and psoriasis [13–16]. Atopic dermatitis results in a dysbiosis that favors expansion of *Staphylococcus aureus*, which correlates with the severity of disease [13, 17–19]. Interestingly, the Th2-based immunologic changes that drive atopic dermatitis share significant similarities with secondary LE and may thus implicate a role for inflammation-driven dysbiosis in LE pathogenesis. However, to determine if observed microbiome changes contribute to infection risk in secondary LE patients, alternative methods for differential abundance analysis are required, such as metagenomic sequencing to clarify the functional profile of the microbes detected [20]. To date, only one previous study [21] has analyzed bacterial dysbiosis and infection risk in LE resulting from filarial infections.

Here, using high-throughput genomic sequencing, we analyzed skin microbiome composition in paired affected/unaffected skin samples from patients with unilateral upper extremity cancer-related LE. We show high compositional heterogeneity in the skin microbiome in LE and that variations in relative abundance relate, in part, to relative limb volume difference between the normal and LE limb. Our results highlight a new area of study for LE pathology.

## 2. Materials and methods

### 2.1 Patient demographics and skin sample collection

Patients were recruited from the Plastic and Reconstructive Surgery Lymphedema Clinic at Memorial Sloan Kettering Cancer Center (MSK). The inclusion criteria were: (1) age >18 years; (2) unilateral upper extremity lymphedema, with a >10% difference in volume and texture of the affected limb; and (3) moderate to severe severity according to the International Society of Lymphology (ISL). Patients with an acute inflammatory condition, such as flu-like illness, skin infection, or fever-associated illness within six weeks of sample collection were excluded. Additional exclusion criteria included: (1) history of metastatic or untreated breast cancer; (2) systemic or topical antibiotic treatment within six weeks of sample collection; (3) history of chronic skin disease or open wounds of the upper extremities; and (4) recent use of antiseptic topical applications. In total, thirty (28 female and 2 male) patients with LE were selected based on the inclusion criteria. All female patients had a primary breast cancer diagnosis. For the two male patients, the underlying diagnoses included squamous cell cancer of the left axilla of unknown primary and left midback melanoma. All patients provided written informed consent. The study was approved by the Memorial Sloan-Kettering Cancer Center’s Institutional Review Board/Privacy Board-A and Institutional Review Board/ Privacy Board-B (IRB 18–536)

Participants were contacted 48 hours prior to sample collection to confirm eligibility according to the inclusion criteria. Twenty-four hours prior to clinic arrival, patients were instructed as follows: (1) do not shower, bathe, or wash forearms with soap or water; (2) avoid creams, moisturizers, perfumes, and lotion applications to the forearms; and (3) limit the use of compressive garments. Skin swabs were collected from the proximal forearm of the normal and LE limbs according to the skin sampling protocol outlined by the Children’s Hospital of Philadelphia (CHOP) Microbiome Center, Division of Gastroenterology, Hepatology, and Nutrition. Briefly, Copan flexible flocked swabs (FLOQSwab®553-C, Copan Diagnostics Inc.) were moistened with sterile PBS, and the volar forearm of the LE limb stroked 60 times, alternating directions vertically and horizontally, over a sampling diameter of <4 cm^2^. The maneuver was repeated for the normal limb using a clean FLOQSwab. Four swabs moistened with sterile PBS alone were collected as negative controls. The swabs were then placed in a dry collection tube, appropriately labeled by paired sample number, and placed in a mobile liquid nitrogen container for overnight shipment to the CHOP Microbiome Center.

### 2.2 DNA extraction, library construction, and 16S rRNA sequencing

DNA sequencing of the bacterial 16S rRNA gene V1-V3 region was carried out at the CHOP Microbiome Center. DNA was extracted using the PowerSoil kit (Qiagen). DNA library preparation was performed using dual-barcoded primers targeting the V1-V3 regions of the bacterial 16S rRNA gene. PCR products were sequenced as 300 base-pair reads using the Illumina MiSeq instrument16S rRNA marker gene sequence data was analyzed using the QIIME2 pipeline (v2019.7) with default parameters [22]. Denoising and selection of the amplicon sequence variants (ASVs) were performed with DADA2 software [23]. Taxonomic assignments were generated using a Naïve Bayes classifier trained on the Greengenes reference database (v13_8) [24].

### 2.3 Statistical analysis

All statistical analyses were performed in R 4.1.1 (R Core Team, 2021). Descriptive statistics for the study population are reported, including median and interquartile range (IQR) for continuous variables and percentages for categorical variables. Missing data were omitted from descriptive statistics. Shannon index and Inverse Simpson index were calculated for microbial α-diversity. Wilcoxon signed-rank test was used to test paired differences of α-diversity between normal and LE samples. Bray-Curtis distance (BCD) and Aitchison’s distance (AD) were calculated for microbial β-diversity. Principal coordinate analysis (PCoA) and corresponding 2D visualization plots were conducted based on BCD matrix. Multivariable linear regression was applied to investigate the association between patient-specific paired microbial distances (BCD or AD) and patient clinical characteristics. Microbial variability analysis was conducted to reveal which taxa had high variation between paired LE and normal samples, where variability was defined as the absolute value of the relative abundance difference between paired samples. Linear decomposition model [25] was applied to test taxa differential abundance between paired LE and normal samples, adjusting for previously described clinical variables. The obtained p-values were adjusted for multiple testing by sequential Monte Carlo multiple testing procedure [26]. P-values <0.05 were considered statistically significant.

## 3. Results

### 3.1 Microbial profiles are similar in normal and LE skin

**Fig 1:**
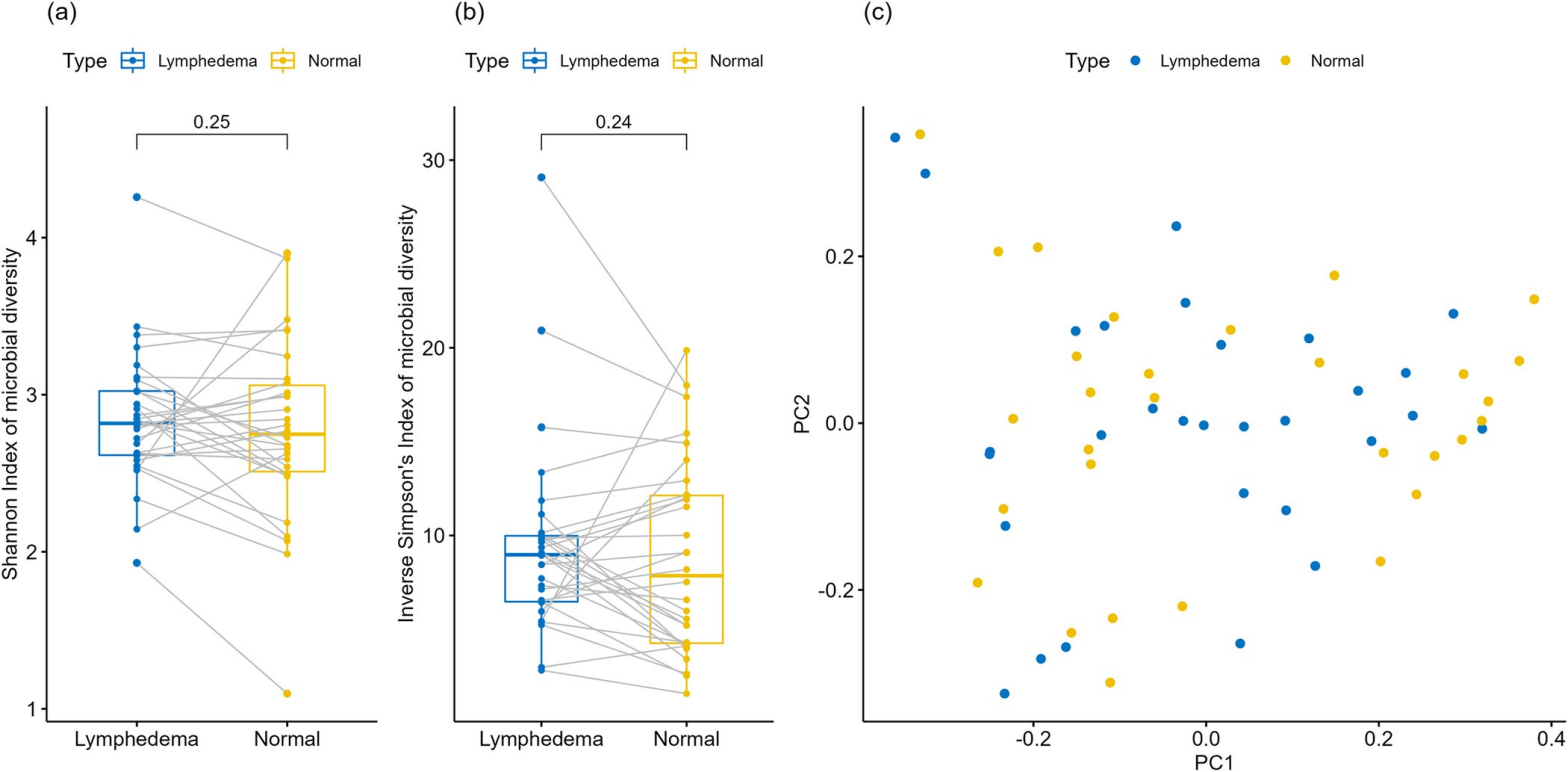
Microbial diversity of normal and lymphedema limbs in 30 patients with upper extremity secondary lymphedema. (a) Boxplot of Shannon index for lymphedema and normal limbs; paired samples are connected by grey lines. P-values obtained by Wilcoxon signed-rank test for paired data. (b) Boxplot of inverse Simpson’s index for lymphedema and normal limbs; paired samples connected by grey lines. P-values obtained by Wilcoxon signed-rank test for paired data. (c) Principal component analysis (PCoA) plot of the first two principal coordinates (PC1, PC2) based on Bray-Curtis distance matrix.

We collected skin swabs from the proximal forearm of the normal and LE limb in 30 patients with unilateral upper extremity LE (**Table 1)**. Analysis by high-throughput 16S RNA sequencing demonstrated no significant difference in microbial α-diversity between normal and LE skin as measured by Shannon and Inverse Simpson’s indices **(Fig 1A and 1B)**. Moreover, there was no consistent difference in α-diversity when comparing paired normal and LE limbs **(Fig 1A and 1B; grey lines)**—some patients had higher diversity in the LE limb, while others had a higher diversity in the normal limb. Consistent with these findings, principal coordinate analysis (PCoA) showed no clear separation of LE and normal samples according to the Bray-Curtis distance (BCD) **(Fig 1C)**.

**Table 1:** Patient demographics and clinical characteristics.

Characteristic	N = 30
Sex	
Female	27 (93%)
Male	2 (6.9%)
Unknown	1
Age (years)	59 (12)
Unknown	1
Race	
African-American	1 (3.6%)
Asian Indian	1 (3.6%)
White	26 (93%)
Unknown	2
History of infection	
No	10 (36%)
Yes	18 (64%)
Duration of LE (months)	82 (55)
Unknown	3
Absolute volume differential	671 (482)
Unknown	4
Relative volume differential (%)	30 (22)
Unknown	4
ISL stage	2.0 (0.0)
Unknown	2

Data are n(%) or Mean (SD)

LE: lymphedema; ISL: International Society of Lymphology

### 3.2 Clinical factors are associated with microbial dissimilarity and heterogeneity in LE

**Fig 2:**
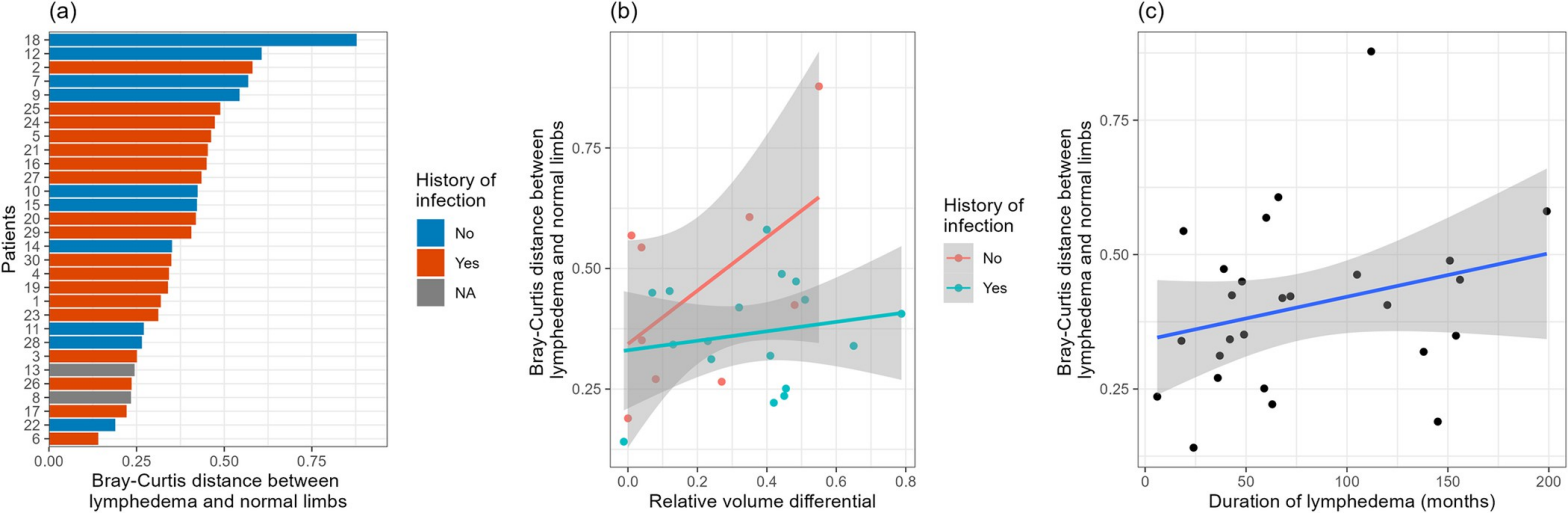
Association of microbial distance between paired limbs and clinical covariates. (a) Swimmer plot of Bray-Curtis distance between lymphedema and normal limbs, with history of infection indicated. (b) Scatter plot of paired Bray-Curtis distance versus relative volume differential, with fitted line and confidence band from marginal linear regression, stratified by history of infection. (c) Scatter plot of paired Bray-Curtis distance versus duration of lymphedema in months, with fitted line and confidence band from marginal linear regression.

We next investigated the degree of microbial heterogeneity between normal and LE limbs. BCD quantifies compositional distance between two samples on a scale of 0 to 1, where distances closer to 0 indicate similar microbial compositions, while distances closer to 1 imply highly different profiles. Overall, there was a high degree of heterogeneity in our cohort **(Fig 2A)**—for example, subject 18 had dramatically different microbial profiles between limbs (BCD = 0.88), while subject 6 had very similar microbial compositions between limbs (BCD = 0.15). In addition, 4/5 patients with the highest paired BCDs had no history of infection.

We used multivariable linear regression to further investigate the association between clinical variables and BCD. Interestingly, we found that increases in limb volume were associated with increased BCD in patients who did not have a history of infection **(Table 2A)**; a 1-fold increase in volume between the LE and normal limbs resulted in a 0.58-unit increase in paired BCD for patients without a history of infection (95% CI = 0.11, 1.05; p = 0.02). In contrast, in patients with a history of infection, a 1-fold increase in limb volume was associated with a non-significant, 0.07-unit increase in paired BCD (95% CI = -0.26, 0.39; p = 0.69). The correlation between relative volume differential and BCD in patients with and without a history of LE-related infection is shown in **Fig 2B.** The duration of LE shows positive but insignificant association with BCD (p = 0.11; **Table 2A** and **Fig 2C)**. The multivariable linear model for the Aitchison’s distance indicated similar associations, where a 1-fold increase in volume between the LE and normal limbs resulted in a significant increase in AD for patients with a history of infection (11.30-unit increase; 95% CI = 0.635, 21.97; p = 0.04) and without (11.23-unit increase; 95% CI = 1.231, 21.22; p = 0.03; **Table 2b**).

**Table 2:** Point estimates, 95% confidence intervals, and corresponding p-values of patient clinical characteristics in the multivariable linear regression model of (a) paired Bray-Curtis distance and (b) paired Aitchison’s distance.

**(a) Model for Bray-Curtis distance**			
Characteristic	Estimate	95% CI	p-value
**History of infection **			
No	—	—	
Yes	-0.043	-0.237, 0.152	0.651
**Duration of LE**	0.001	0.000, 0.002	0.112
**Relative volume differential **			
No history of infection	0.580	0.111, 1.048	**0.018 **
History of infection	0.066	-0.257, 0.388	0.689
**(b) Model for Aitchison’s distance**			
Characteristic	Estimate	95% CI	p-value
**History of infection**			
No	—	—	
Yes	4.403	-4.672, 13.48	0.323
**Duration of LE**	0.087	-0.011, 0.185	0.078
**Relative volume differential**			
No history of infection	11.30	0.635, 21.97	**0.039**
History of infection	11.23	1.231, 21.22	**0.028**

CI: confidence interval

### 3.3 Taxa-specific analysis demonstrates high genus-level variability between normal and LE limbs

**Fig 3:**
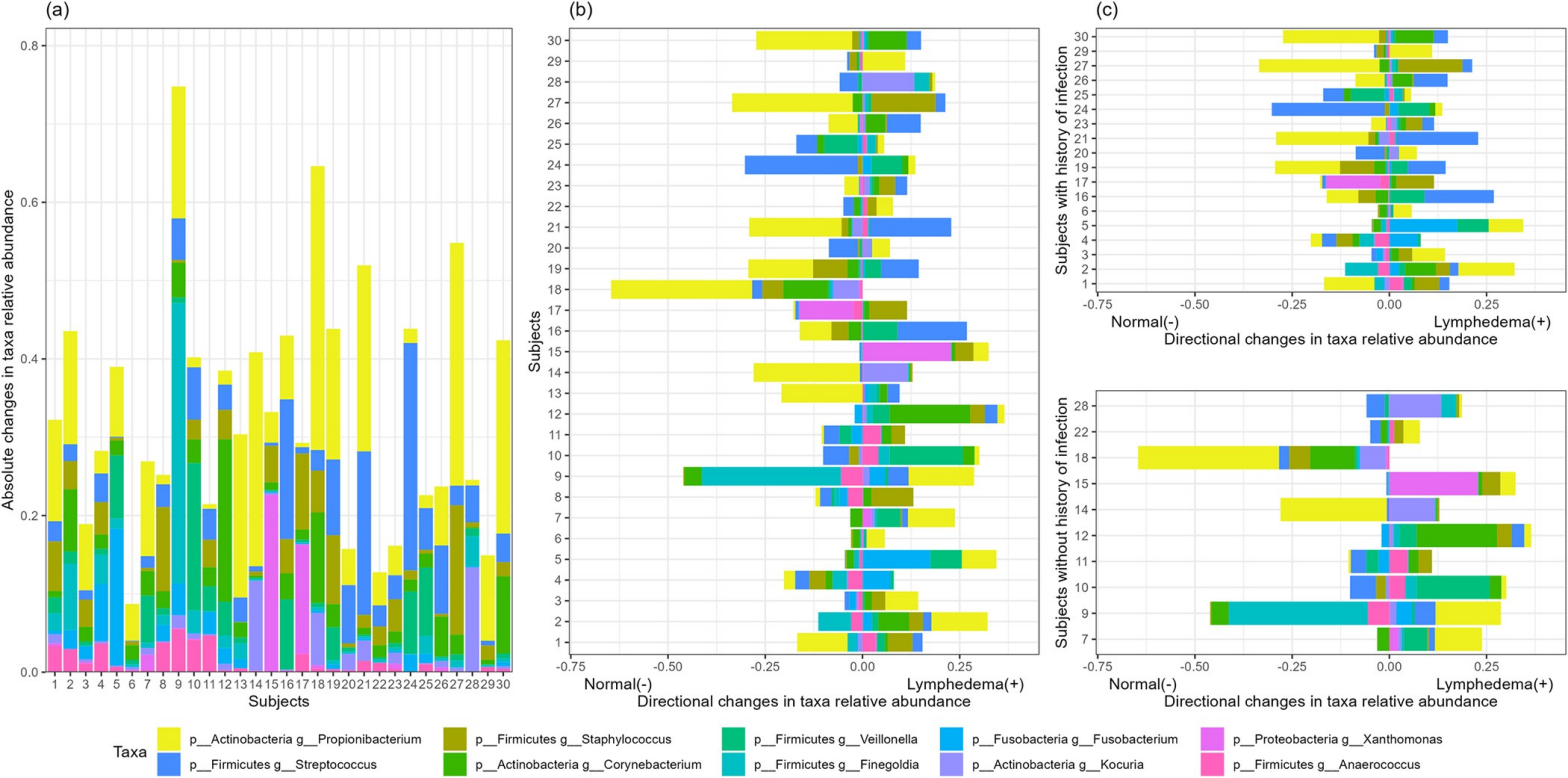
Patient-specific taxa variability between normal and lymphedema limbs for the ten most variable taxa. (a) Stacked bar chart of patient-specific taxa variability, defined as the absolute difference of taxa relative abundance. (b) Directional bar chart of patient-specific difference of taxa relative abundance. (c) Directional bar chart of patient-specific difference of taxa relative abundance, stratified by history of infection. p: phylum; g: genus; c:class.

Across all samples, 872 genus-level bacterial taxa were identified. To identify taxa with the highest variation between paired normal and LE samples, we calculated the absolute difference of taxa relative abundance between each LE and normal pair (**Table 3 and Fig 3A**). In particular, the genera *Propionibacterium* and *Streptococcus* demonstrated high variations between paired limbs, with average variability in relative abundance of 10% and 5%, respectively. The microbial variability also varied across patients, further indicating high heterogeneity among LE patients. On the other hand, the direction of relative abundance changes was not uniform across patients (**Fig 3B**), where the average abundance differences were close to zero (**Table 3**). Moreover, no obvious directional shifts were observed for these highly unstable taxa for patients with differing histories of infection (**Fig 3C**). Finally, taxa differential abundance testing [25] also showed that no taxa were significantly differentially abundant between normal and LE after adjusting for potentially confounding clinical variables and false discovery rate [26] (**Tables 3 and 4**). Mean variability was calculated as average absolute difference of taxon-specific relative abundance between paired normal and LE samples. Mean differential abundance was calculated as average signed difference of taxon-specific relative abundance between paired normal and LE samples. Taxa direction indicates the limb with the higher abundance.

**Table 3:** Top 10 variable taxa found in microbial variability analysis.

Top ten most variable taxa (p: phylum; g: genus; c: class)	Mean Variability	Mean Differential Abundance	Taxa direction Normal (-) Lymphedema (+)	Unadjusted p-value	Adjusted p-value
p__Actinobacteria g__Propionibacterium	0.105	-0.041	**- **	0.237	0.793
p__Firmicutes g__Streptococcus	0.052	0.004	**+ **	0.702	0.893
p__Firmicutes c__Bacilli	0.036	0.009	**+ **	0.540	0.793
p__Firmicutes g__Staphylococcus	0.035	0.013	**+ **	0.418	0.793
p__Actinobacteria g__Corynebacterium	0.033	0.008	**+ **	0.394	0.793
p__Firmicutes g__Veillonella	0.028	0.018	**+ **	0.038	0.793
p__Firmicutes g__Finegoldia	0.024	-0.014	**- **	0.479	0.793
p__Fusobacteria g__Fusobacterium	0.016	0.008	**+ **	0.822	0.921
p__Actinobacteria g__Kocuria	0.015	0.007	**+ **	0.780	0.905
p__Proteobacteria g__Xanthomonas	0.014	0.003	**- **	0.480	0.793

**Table 4:** Top 10 genera with smallest unadjusted p-values obtained by the linear decomposition model (LDM) test of differential abundance for paired data.

Genera with unadjusted p < 0.05 (p: phylum; g: genus)	Mean Differential Abundance	Taxa direction Normal (-) Lymphedema (+)	Unadjusted p-value	Adjusted p-value
p__Proteobacteria g__Methylobacterium	-0.006	**- **	0.002	0.793
p__Proteobacteria g__Janthinobacterium	-0.001	**- **	0.012	0.793
p__Actinobacteria g__Arthrobacter	-0.000	**- **	0.013	0.793
p__Proteobacteria g__Rhodoplanes	-0.000	**- **	0.017	0.793
p__Proteobacteria g__Enhydrobacter	-0.002	**- **	0.028	0.793
p__Proteobacteria g__Sphingomonas	-0.004	**- **	0.031	0.793
p__Proteobacteria g__Pseudomonas	-0.001	**- **	0.034	0.793
p__Proteobacteria g__Caulobacter	-0.000	**- **	0.037	0.793
p__Firmicutes g__Veillonella	0.018	**+ **	0.038	0.793
p__Proteobacteria g__Brevundimonas	-0.008	**- **	0.039	0.793

## 4. Discussion

A better understanding of the etiology and pathogenesis of LE is critical for developing novel treatment modalities aimed at a cure for the 1 in 1000 Americans affected by the disease [27]. Although LE appears to be mediated by a predominant Th2 inflammatory response, it is evident that a combination of intrinsic and extrinsic factors plays a role in disease development and progression. In this study, we utilized high-throughput sequencing to investigate the role of the skin microbiome in LE pathophysiology. We found that differences in microbial composition of the normal and LE limb is heterogeneous among patients with varying histories of infection and is related to relative limb volume changes between limbs.

Bacterial dysbiosis, or a disruption in the balance of resident microbes, has been implicated in a variety of cutaneous diseases [28]. Particularly, in atopic dermatitis, a loss of microbial diversity is associated with disease severity [13]. Similarly, in filarial LE, the most common form of secondary LE worldwide caused by *Wuchereria bancrofti* infection, an increase in *Staphylococcal aureus* is observed in filarial skin when compared to skin of healthy controls [21]. In contrast to the former study, our study aimed to characterize the bacterial skin microbiome in individuals with non-filarial upper extremity secondary LE. Secondly, whereas the previous group relied on culture and mass spectrometry techniques to draw associations between skin commensal diversity and infection risk, we utilized high-throughput sequencing technology for taxa identification, which reduces the risk of underestimating or misidentifying the species present in a sample [21, 29]. An additional strength of our study, the paired sample study design, accounts for confounders that may otherwise be present when comparing to healthy controls.

Our findings indicate that multiple genera of the phyla Firmicutes demonstrate high variability between normal and LE samples (**Table 3**). Specifically, the most variable genera observed in the LE limb included *Streptococcus*, *Staphylococcus*, *Veillonella*, *Fusobacterium*, *and Anaerococcus*. The microbial variability analysis performed in this study is inspired by the concept of microbial volatility, which describes the temporal instability of the microbiome [30, 31]. Traditionally, volatility has been studied in the context of the gut microbiome, particularly as it relates to inflammatory bowel diseases [32]. Interestingly, observed volatility in intestinal physiology has been shown to influence inflammatory activity at distant organ sites, namely the skin barrier, leading to this concept of the gut-skin axis [32, 33]. The gut-skin axis, or the involvement of the gut microbiome in regulating health and disease states of the skin, has been linked with the development of chronic inflammatory skin conditions, such as psoriasis, rosacea, and acne [33]. Disturbances in the gut microbiome may contribute to the microbial variability that we observe between patients with LE. However, further studies investigating the gastrointestinal health of patients that develop disease is warranted if a bidirectional relationship between gut dysbiosis and LE development is to be established.

More recently, volatility has been studied in the context of microbial variations in response to elevated levels of stress, which is relevant to the microbiome-gut-brain axis [30]. Bastiaanssen and colleagues observed significant positive correlations between chronic psychosocial stress and the degree of gut microbiome volatility in mice and humans. They speculate that hosts with the most volatile microbiomes are most susceptible to stress-associated symptoms. Although no causal link has been established between stress levels and LE development, chronic stress has been recognized as a barrier to effective management of LE [34]. Notably, in a single-center clinical trial evaluating the effect of combined psychosocial relaxation techniques and comprehensive decongestive therapy (R-CDT) to comprehensive decongestive therapy (CDT)alone on depression scores and the volume of edema in 31 patients with breast cancer-related lymphedema, a significant reduction in depression scores (p = 0.024) and a downtrend in mean edema volume at 9-week follow-up (p = 0.470) was observed in the RCDT group when compared to CDT alone [35]. Taken together with the findings from our current study, it is possible that the variability observed between paired samples is influenced by psychosocial stress levels and that limb volume changes may, in part, contribute to microbial composition detected in skin swabs. Given the dynamic nature of the microbiome and the fact that our samples were taken at a single timepoint, our observations of paired sample variability may not completely reflect the temporal variations of the microbial communities analyzed.

We also utilized multivariable statistical modeling to study how clinical factors may influence microbial composition between normal and LE limbs. Our results indicate a significant association between microbial distances (BCD and AD) and relative limb volume differential. The accumulation of lymph fluid can alter skin integrity and facilitate the entry of external pathogens; thus, this observation supports a likely relationship between the degree of arm swelling and bacterial populations present on the skin [36, 37]. One could hypothesize that the gradual increase in limb swelling over time observed in LE is in part related to a disruption of the bacterial microbiota. However, because we did not observe any consistent changes in the microbial composition, it is unclear if dysbiosis in favor of a single genera can serve as a marker for the disease.

Kwarteng et al determined that seasonal variations in the microbiota, favoring a shift towards an over-population of *Staphylococcus aureus* is present in filarial lymphedema lesions. They speculate that the observed dysbiosis in combination with a diminished local skin immune system influences the infectious attacks that are frequent to this population. Additionally, their study and others demonstrate that topographical location on the body is a defining factor of bacterial diversity [38, 39]. In our study, skin swabs were obtained from the volar forearm of the LE and the normal limbs. Compared to other areas of the upper limb, the volar forearm is known to harbor a diverse microbial community, making it an ideal region for comparative sequencing studies at symmetric sites [38, 40, 41]. An interesting investigation would be to compare the microbial composition of the LE limb in areas where edema is most apparent and likely correlates with a weakened skin barrier. Using indocyanine green (ICG) lymphography, a minimally invasive diagnostic tool that shows patterns of dermal backflow, may help facilitate this type of study [42, 43].

In recognizing study limitations, the small sample size of 30 patients limited the statistical power of testing taxa differential abundance. Additionally, including multiple timepoints for skin swab analysis and varying locations for sample collection along the lymphedema limb would enhance the robustness of this study. In addition, although 16S amplicon sequencing is a standard approach for characterizing the taxonomic profile of the microbiome in LE, utilization of shotgun metagenomics technology would better discriminate those bacterial communities that play a functional role in the disease process [44, 45]. Future studies with larger sample sizes will allow deeper investigation of differential abundance, patient heterogeneity, and longitudinal dynamics of the microbiota associated with lymphedema. In conclusion, 16S rRNA microbiome sequencing shows high compositional heterogeneity in the skin microbiome between the normal and diseased limbs among patients with upper extremity secondary LE. We encourage further studies into host-microbiome interactions in secondary LE, with a focus on the implications for LE diagnosis and management.

## Supporting information

S1 Table Genus-level relative abundance data for paired lymphedema and normal samples. (XLSX)
